# Physical Activity and Urinary Sodium Excretion Circadian Rhythm: A Population-Based Cross-Sectional Pilot Study

**DOI:** 10.3390/jcm13164822

**Published:** 2024-08-15

**Authors:** Martina Zandonà, Jakub Novotny, Maria Luisa Garo, Ettore Sgro, Rosaria Del Giorno, Luca Gabutti

**Affiliations:** 1 Clinical Research Unit, Department of Internal Medicine, Ente Ospedaliero Cantonale, 6500 Bellinzona, Switzerland; 2 Faculty of Biomedicine, University of Southern Switzerland, 6900 Lugano, Switzerland; jakub.novotny@usi.ch; 3 Biostatistic Unit Mathsly Research, 00128 Rome, Italy; marilu.garo@mathsly.it; 4 Department of Internal Medicine, Ente Ospedaliero Cantonale, 6500 Bellinzona, Switzerland; ettore.sgro@eoc.ch; 5 Angiology Service, Ente Ospedaliero Cantonale, 6500 Bellinzona, Switzerland; rosaria.delgiorno@eoc.ch; 6 Family Medicine Institute, University of Southern Switzerland, 6900 Lugano, Switzerland

**Keywords:** physical activity, hypertension, dipping, circadian pattern, circadian rhythm, sodium, sodium excretion, salt intake

## Abstract

**Background/Objectives:** Blood pressure (BP) is characterized by a circadian rhythm (Circr) with lower nighttime values, called dipping. Non-dipping is associated with higher CVD risk. The Circr of urinary sodium excretion (NaCle), peaking during the day, is linked to BP patterns. Physical activity (PA) is known to improve BP control and enhance the dipping phenomenon, but its possible effect on NaCle remains unclarified. This study aimed to investigate the correlation between PA and the Circr of NaCle and to determine if the relationship is independent of age, sex, BP values, dipping pattern, and salt intake. **Methods:** A pilot cross-sectional analysis was conducted using data from the Ticino Epidemiological Stiffness Study, involving 953 participants in Switzerland. Data collection included standardized questionnaires, blood samples, 24 h urine collections, and ambulatory BP monitoring. Participants were categorized into sedentary, partially active, and active. The effect of PA, NaCl intake, and dipping on the day/night NaCle ratio was assessed with multivariable linear regressions. **Results:** Participants’ median age was 49 years, with 78% having normal BP values and 47% exhibiting a dipping pattern; 51% were classified as sedentary and 22% as partially active. The median NaCl intake was 7.9 g/day. The youngest subjects had a higher hourly NaCle ratio compared to older subjects. Higher NaCl intake correlated with increased BP, a phenomenon more pronounced in men and younger subjects. The hourly day/night NaCle ratio positively correlates with dipping; however, PA did not show a significant correlation with the NaCle ratio. **Conclusions:** This study indicates that while the day/night NaCle ratio correlates with the dipping pattern, PA is unrelated to the circadian rhythm of renal sodium handling. The beneficial effects of PA on BP and cardiovascular health thus appear to be mediated through mechanisms other than NaCle. These are explorative findings only but relativize the need for further investigations on the topic.

## 1. Introduction

Hypertension is one of the main risk factors contributing to the onset of cardiovascular diseases (CVDs) [1,2]. In people with a usual sleep–wake pattern, blood pressure (BP) is characterized by a circadian rhythm, with the highest values during daytime and the lowest during nighttime while asleep [3,4,5]. This decrease in BP values during nighttime is called “dipping”, and it is characterized by a lowering of nighttime pressure of 10–20% compared to daytime BP [6]. The circadian rhythm of blood pressure is usually assessed using ambulatory BP monitoring (ABPM), which measures BP at preset intervals during a 24 h period [3]. Despite the fact that the dipping pattern is physiological, some people have “non-dipping” blood pressure profiles, with a reduction in BP during nighttime <10%, and in some cases “reverse dipping” profiles, with an elevation of BP values during the night. The absence of dipping is associated with increased CVD risk and more serious target-organ damage like left ventricular hypertrophy, microalbuminuria, and cerebrovascular disease [7,8,9,10,11].

It has been suggested that the absence of nocturnal dipping in some subjects could be related to high sodium intake and/or salt sensitivity [12,13]. Furthermore, more recent studies have hypothesized that the non-dipping pattern could be explained by an impaired capacity to excrete enough sodium during the daytime [14,15]. To this point, our group has previously demonstrated that impaired daytime sodium excretion capacity is associated with a reduced dipping pattern in subjects older than 50 years [16].

Urinary sodium excretion is also characterized by a circadian rhythm, with the maximum level of excretion during daytime and the minimum during nighttime [17]. This pattern, and in particular nighttime sodium excretion, is closely linked to blood pressure. Previous findings have in fact suggested that BP represents the primary determinant of sodium excretion during the night. This indicates that an impaired capacity to excrete sodium during the daytime is linked to increased nocturnal BP in order to promote sodium excretion through the pressure-natriuresis mechanism, resulting in the typical non-dipping pattern [17,18,19]. Another important determinant of sodium and water renal excretion regulation is the renal sympathetic nerve activity (RSNA), whose rise promotes renin secretion and renal tubular sodium reabsorption, resulting in sodium retention and increase in BP [20].

Besides blood pressure, age is one of the most important determinants of sodium homeostasis. Aging has been shown to be linked to both salt retention and salt sensitivity and consequently to hypertension [12,13]. With advancing age the kidney undergoes structural and functional changes, including reduction in renal blood flow and glomerular filtration rate [21]; it has also been proved that people 40 years old or older excrete less sodium after saline loading than those younger than 40 years [22]. As an escape mechanism to rebalance extracellular volume and ultimately reduce BP, pressure natriuresis, a rise in sodium excretion due to a pressure related secondary increase in renal perfusion, intervenes physiologically. Still, due to an age-related decline in the contra-regulation of sodium excretion, the net result is an increased prevalence of salt-sensitive hypertension in the older population [23].

Age is also related to a decrease in nocturnal BP fall [24], a phenomenon that could be explained by the loss of the sleep-dependent ability to reduce sympathetic vascular tone and by the presence of vascular wall changes leading to stiffness [25].

Regular physical activity (PA) is a key protective factor for the prevention and management of many chronic diseases like CVD, type 2 diabetes, cancer, obesity, depression, and osteoporosis [26,27]. Engaging in regular exercise is known to reduce blood pressure (BP) [28] and stimulates physiological adaptations for general health improvements [29]. Current recommendations from the American College of Sports Medicine suggest that most individuals with hypertension should perform 90–150 min/week of moderate intensity aerobic exercise [30].

As far as the circadian pattern of blood pressure, physical activity is associated with BP dipping during the nighttime, and previous studies showed that the non-dipping pattern could be improved by long-term aerobic exercise training [31,32].

Taking into consideration the relationships between the circadian rhythm of urinary sodium excretion and the dipping pattern of blood pressure and between the latter and physical activity, and knowing that both BP, PA and Na metabolism are related to common mechanisms (sympathetic system [33], cortisol pathway [34], renin–angiotensin–aldosterone system [35,36]), we hypothesized that PA could independently influence sodium excretion. Therefore, the aim of this pilot study is (i) to investigate a possible correlation between physical activity and the circadian rhythm of urinary sodium excretion and (ii) to assess if this influence is independent from age, sex, blood pressure values, dipping pattern, and salt intake.

## 2. Materials and Methods

### 2.1. Study Design

The present pilot study is based on a cross-sectional analysis of a population-based research project, the “Ticino Epidemiological Stiffness Study, TEST-study”, which took place between the years 2017 and 2018. It was carried out in the Italian-speaking part of Switzerland (Canton Ticino) and involved residents aged ≥ 18 years. Participants were recruited with a random sampling from a mailing list that was provided by the Swiss Federal Statistic Department, with a response rate of 86%.

### 2.2. Ethical Approval

The TEST-study was performed in conformity with the 1964 Helsinki Declaration and its subsequent revisions. It was approved by the Swiss ethics committee (CE 3115-2016-01718) and all participants provided a written informed consent to take part in the study.

### 2.3. Population Sample

A total of 1202 participants were recruited in the original study. Each subject was administered a standardized questionnaire exploring health, pathological case history, eating habits, and physical activity; blood samples were collected in order to analyze serum glucose, HbA1c, creatinine, low-density lipoprotein cholesterol, high-density lipoprotein cholesterol, triglycerides, total cholesterol, and cystatin. A 24 h urine collection, divided in “daytime” and “nighttime” based on subject’s self-reported bed-time and wake-up time, was performed in order to collect different samples from the prevalent sitting/standing position, and the supine one. To reduce the risk of bias due to the different duration of “day” and “night” in the subjects and given the well-known intra-individual variability in urine collection accuracy, the sodium excretion was calculated using the hourly urinary creatinine excretion as time reference [37].

The urine samples were analyzed for sodium and creatinine concentrations. Invalid collections with less than 400 mL or less than 2.5 mmol of urinary creatinine or more than 6000 mL or 30.0 mmol of urinary creatinine in the 24 h urine and participant reported losses of more than 100 mL urine were excluded from the study. Concerning blood pressure measurements, every participant was equipped for 24 h with an Ambulatory Blood Pressure Monitor (ABPM). The device recorded measurements every 30 min during the day and once every hour during the night. The blood pressure was recorded during working days and the participants were asked to maintain their usual routine [11]. The blood pressure monitoring was performed the same day of the urine collection. 

**Figure 1:**
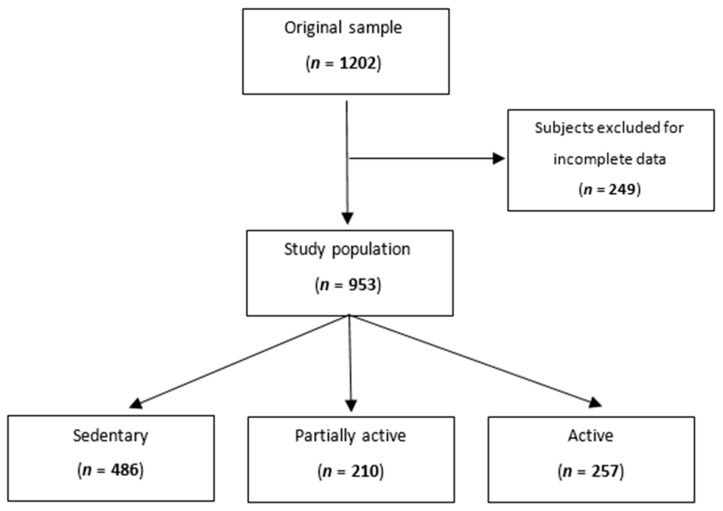
Flowchart showing the participants’ selection procedure.

After excluding subjects with missing data for total sodium excretion, level of physical activity, blood pressure measurements, or urine collection, we analyzed 953 participants (Figure 1).

Based on the questions exploring levels of physical activity (minutes of walking per day, physical activity at work, and heavy physical activity in a week) in the standardized questionnaire, participants were divided into three categories of “physical activity level”: “sedentary”, “partially active”, and “active”.

The participants were considered to have risk factors when one or more of the following characteristics were present: current smoker, diabetes, previous cardiovascular disease, chronic kidney disease (CKD) stage 3 or more as per KDIGO classification, LDL (low-density lipoprotein) ≥ 4.4 mmol/L, hypertension, use of hypolipidemic and/or antihypertensive drugs, metabolic syndrome. 

According to the NCEP ATP III definition, metabolic syndrome was diagnosed if three or more of the five key criteria were met: waist circumference > 101.6 cm (men) or 88.9 cm (women), blood pressure > 130/85 mmHg, fasting triglyceride level > 1.69 mmol/L, fasting high-density lipoprotein (HDL) cholesterol level < 1.03 mmol/L (men) or 1.29 mmol/L (women), and fasting blood glucose over 5.5 mmol/L [38]. In case of unavailability of a fasting blood glucose, a HbA1c value of 5.7% was used as the cut-off [39]. eGFR was calculated with the CKD-EPI Creatinine-Cystatin Equation (2021).

The circadian rhythm of sodium excretion was calculated using the ratio between the hourly sodium excretion during the day by the hourly sodium excretion during the night.

The 24 h NaCl intake was estimated from the 24 h urinary sodium excretion considering that 1 mmol of sodium corresponds to 0.0584 g of NaCl and assuming that most of the Na is ingested as a chloride salt.

### 2.4. Statistical Methodology

Preliminarily, a factorial analysis was conducted to identify the possible underlying dimensions in the group of variables related to physical activity. The identification of the factors derived from the individual scales and the interpretation of the factor loadings (saturation) was performed by setting the value 0.50 as the lower limit of acceptability of the individual item. Factor identification was performed after applying a varimax rotation to maintain the independence of the factors [40,41]. To understand the suitability of the data for factor analysis, the Kaiser–Meyer–Olkin test was performed [42]. In this way, two factors, one related to daily physical activity and the other to heavy physical activity, were identified and used to calculate a physical activity score (results not reported). Therefore, subjects were classified as sedentary, partially active, or active (Table 1).

Due to the non-normal distribution of the quantitative variables, descriptive statistics were expressed as median and interquartile ranges (25th–75th) (IQR). Categorical data were presented as relative frequencies and percentages. Comparisons among groups were performed using non-parametric tests, Mann–Whitney U test or Kruskal–Wallis test, with the latter followed by the Dunn procedure to determine statistically significant two-by-two comparisons. In addition, the chi-square test was used for categorical variables. Multivariable linear regressions were performed in the main sample and by subgroups (i.e., sex, age range, blood pressure level, and risk factors) to assess the effects of physical activity, daily NaCl intake, and dipping on the ratio. The statistical significance level was set at 0.05 (*p* < 0.05). Statistical analysis was performed using STATA18 (StataCorp., College Station, TX, USA).

## 3. Results

### 3.1. Descriptive Statistics

Nine hundred and fifty-three patients with a median age of 49 years (IQR: 41–57) were included in the study (Table 2). More than 78% of the subjects had normal blood pressure values, and almost half of the subjects had a drop in blood pressure of 10% or more between day and night. More than 57% of the subjects had three or more of the following risk factors: smoking, diabetes, previous cardiovascular disease, chronic kidney disease (CKD) ≥ 3, LDL ≥ 4.4 mmol/L, hypertension, taking hypolipidemic drugs, taking antihypertensive drugs, metabolic syndrome.

Approximately 50% of subjects had a sedentary lifestyle, while the remaining 22% and 27% were classified as partially and fully physically active, respectively. Specifically, less than 3% of patients reported walking time less than 10 min daily. The ratio of hourly urinary Na excretion had a median of 1.2 Na(g)/Na(n) (IQR: 0.9–1.6). The median NaCl intake was 7.9 g/day (IQR: 5.7–10.4), while the mean NaCl intake was 8.3 ± 3.3 g/day (women: 7.3 ± 2.9 g/day, men: 9.5 ± 3.3 g/day). eGFR was 119.2 mL/min/1.73 m^2^ (IQR: 111.2–125.4) in subjects <40 years, 106.3 mL/min/1.73 m^2^ (IQR:98.3–114.8) in subjects 40–65 years and 88.1 mL/min/1.73 m^2^ (IQR 76.1–98.4) in subjects ≥65 years (*p* < 0.001).

**Figure 2:**
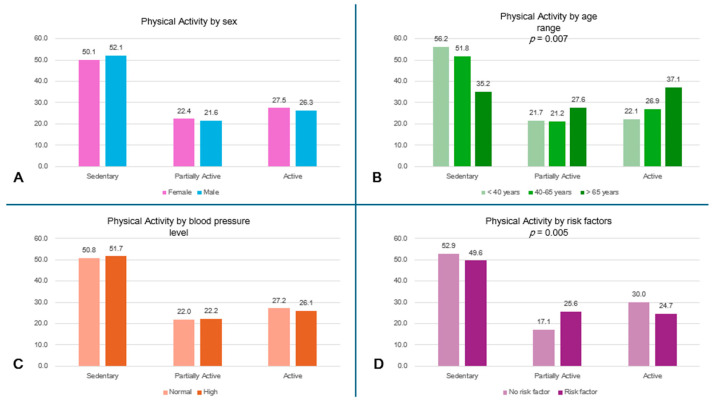
Physical activity level by subgroups. (**A**) Physical activity level by sex. (**B**) Physical activity level by age range (*p* = 0.007). (**C**) Physical activity level by blood pressure. (**D**) Physical activity level by risk factors (*p* = 0.005).

More than 55% of the subjects <40 years and 50% of the entire population led a sedentary lifestyle, while the oldest subjects had a higher level of physical activity (*p* = 0.007) (Figure 2A). A high percentage of subjects without risk factors (52.9%) reported sedentary behaviors; the comparison in physical activity levels between subjects with and without risk factors is shown in Figure 2D.

**Figure 3:**
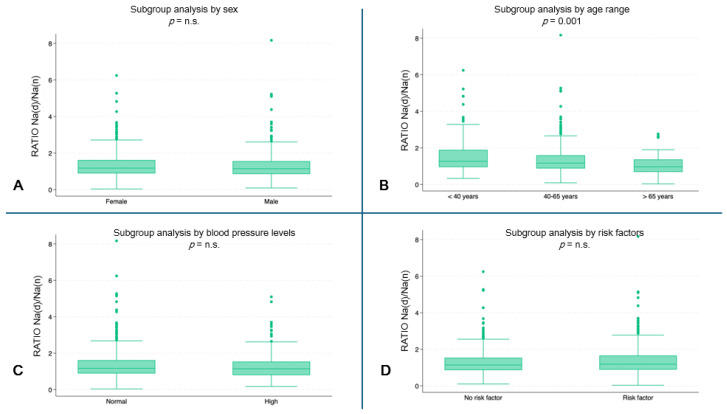
Hourly ratio of Na excretion (day/night) by subgroups. (**A**) Ratio by sex. (**B**) Ratio by age range (*p* = 0.001). (**C**) Ratio by blood pressure. (**D**) Ratio by risk factors. (n.s. = not significant).

The youngest subjects showed a higher ratio of hourly Na urinary excretion (median 1.3, IQR: 0.9–1.9) compared to the oldest age groups (40–65 years: median 1.2, IQR: 0.8–1.6; ≥65 years: median: 0.9, IQR: 0.7–1.3) (*p* = 0.001) (Figure 3B). No statistically significant differences were found in the other subgroup analyses (Figure 3A,C,D).

**Figure 4:**
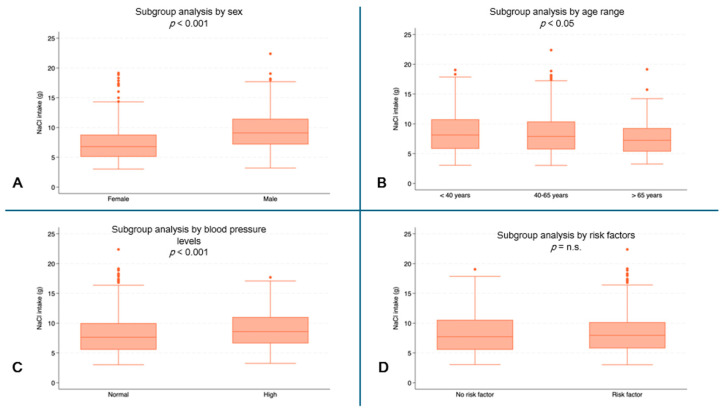
NaCl intake (g) by subgroups. (**A**) NaCl consumption (g) by sex (*p* < 0.001); (**B**) NaCl consumption (g) by age range; (**C**) NaCl consumption (g) by blood pressure (*p* < 0.001); (**D**) NaCl consumption (g) by risk factors. (n.s. = not significant).

Male subjects reported higher values for daily NaCl intake (median: 9.1 g, IQR: 7.2–11.4) than female subjects (median 6.8 g, IQR: 5.1–8.8, *p* < 0.001) (Figure 4A). Higher NaCl intakes were observed in the youngest patients (median 8.1 g, IQR: 5.8–10.8) than in the oldest patients (median: 7.2 g, IQR: 5.4–9.3, *p* = 0.0076) (Figure 4B). Higher values of NaCl intake were also recorded in subjects with high blood pressure (median: 8.6 g, IQR: 6.6–11.0) compared to subjects with normal blood pressure (median: 7.6 g, IQR: 5.5–10.0, *p* < 0.001) (Figure 4D).

**Figure 5:**
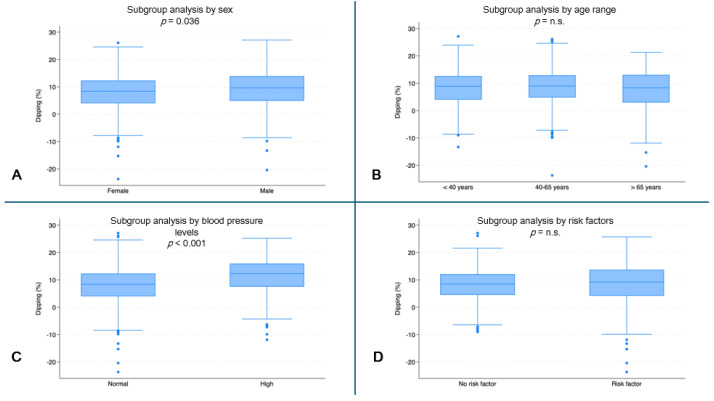
Systolic dipping (%) by subgroups. (**A**) Dipping (%) by sex (*p* = 0.036). (**B**) Dipping (%) by age range. (**C**) Dipping (%) by blood pressure (*p* < 0.001). (**D**) Dipping (%) by risk factors. (n.s. = not significant).

Female subjects had the lowest dipping values (median: 8.4%, IQR: 4.0–12.3) compared to male subjects (median 9.6%, IQR: 4.9–13.9, *p* = 0.036) (Figure 5A). Subjects with normal blood pressure had lower dipping values (median: 8.4%, IQR: 4.0–12.3) than subjects with high blood pressure (median: 12.3%, IQR: 7.5–16.0, *p* < 0.001) (Figure 5C).

### 3.2. Multivariate Analysis

Overall, the multivariate analysis showed that an increase of 1% in dipping resulted in a 0.01 Na(d)/Na(n) (95%CI: 0.01–0.02) increase in the ratio (Table 3).

Subgroup analysis revealed that dipping had a positive significant effect on the ratio in both female (β = 0.01, 95%CI: 0.01–0.02, *p* = 0.004) and male (β = 0.02, 95%CI: 0.01–0.03, *p* = 0.004) subjects (Table 4A), in subjects older than 40 years (40–65 years: β = 0.02, 95%CI: 0.01–0.02, *p* = 0.001; ≥65 years: β = 0.02, 95%CI: 0.01–0.03, *p* = 0.005) (Table 4B), in subjects with and without high blood pressure (normal: β = 0.01, 95%CI: 0.01–0.2, *p* = 0.019; high: β = 0.04, 95%CI: 0.02–0.06, *p* < 0.001) (Table 5A), and in subjects with risk factors (β = 0.02, 95%CI: 0.01–0.03, *p* = 0.001) (Table 5C). In the oldest subjects, NaCl intake also showed a positive effect on the ratio, so an increase in daily NaCl intake by 1 g could be related to an increase in the ratio values by 0.06 Na(d)/Na(n) (95%CI: 0.02–0.09, *p* = 0.001) (Table 4B).

## 4. Discussion

Blood pressure is characterized by a circadian rhythm, with nighttime values 10–20% lower compared to daytime [6]. This reduction in BP is called dipping and its absence is associated with increased CVD risk. Previous findings suggest that the non-dipping pattern could be at least partially explained by an impaired capacity to excrete, during the day, enough of the sodium intake. Urinary sodium excretion is in fact in turn characterized by a circadian rhythm, with higher hourly excretion levels during the day and lower during the night. Circumstances leading to insufficient sodium excretion during the day can produce an increase in nocturnal BP, resulting in a compensation of the sodium excretion through the pressure-natriuresis mechanism. Both BP levels and the urinary sodium excretion pattern are influenced by age, with a tendency to daytime salt retention and to a tamped nocturnal BP fall. Regular physical activity plays an important role for the prevention of CVD and is associated with a more pronounced BP dipping. Physical activity, BP, and sodium metabolism are influenced by common mechanisms and signaling pathways, such as the sympathetic system, the hypothalamic–pituitary–adrenal axis and the renin–angiotensin–aldosterone system. Taking into consideration the relationship between the circadian rhythm of urinary sodium excretion and the dipping pattern of blood pressure and between the latter and physical activity, this study tried to explore whether physical activity could independently influence the pattern of urinary sodium excretion.

The population examined in this study showed a prevalence of sedentary or partially active lifestyles, with the subjects over 65 being more active than the ones in the other age ranges. These results seem to be in contrast with the data reported by the “Switzerland Physical Activity Factsheet 2019”, according to which sufficient physical activity levels were present in over 74% of people under 65 years and in 72% of subjects ≥65 years [43]. Our results could be explained by the fact that older people who decided to take part in the study could have been healthier and more active than the general population, producing a selection bias. Based on the results, we might, however, suspect a reversal of the traditional social dynamics, where younger people were assumed to have higher activity levels. This could be explained by an increased awareness in older people of the importance of lifestyle in maintaining health, translating into a greater willingness to be physically active, and by the increasing number of programs and resources for the elderly available in Switzerland. On the other hand, this trend, if an expression of the truth, raises major concerns for younger people, who may become at increased risk of cardiovascular disease. 

Concerning BP, using their values as a categorical variable (normal vs. high), we did not find a significant difference in the three groups of physical activity levels. 

The daily mean salt consumption (8.3 ± 3.3 g of NaCl/day; men: 9.5 ± 3.3 g/day 7.7 g; women: 7.3 ± 2.9 g/day) was a little lower than expected, taking into consideration that the “Swiss survey on salt intake” showed a mean salt consumption of 10.5 g for men and 9.0 g for women [44]. Explanations could be the different sample sizes, with the Swiss salt study enrolling only 216 participants from our region, the time elapsed between the two studies (7 years), and the effect of the Swiss campaigns to curb salt consumption (2008–2012 and 2013–2016). As expected, and shown in previous studies and mostly attributed to a higher food consumption, we found that sodium intake was higher in men compared with women. 

The daily salt intake was related to the hourly urinary sodium excretion ratio only in older people. This correlation could be explained by the fact that older people tend to have impaired Na excretion and/or greater salt sensitivity [21,45,46]. As suggested by the literature, and for the same reason, the hourly urinary sodium excretion ratio result was higher in the younger population. 

Patients with normal BP resulted in having lower dipping values than patients with high blood pressure. This counterintuitive result is explained by the fact that our analysis did not separate subjects treated with anti-hypertensive drugs from those without. In fact, 12.0% (*n* = 89) of subjects with normal BP had ongoing anti-hypertensive treatment, and people with a diagnosis of hypertension, even if treated, often show a non-dipping pattern. Furthermore, as expected, a higher sodium intake was seen in people with hypertensive values [46]. 

The extent of dipping was positively correlated to the hourly urinary sodium excretion ratio in both sexes and in subjects ≥40 years, confirming that a physiological circadian rhythm of BP is related to a similar pattern of sodium metabolism, with higher levels of urinary Na excretion during the day. The lack of correlation in subjects <40 years could be explained, as told before, by the fact that younger people are less inclined to translate an increase in salt intake into a rise in BP [19]. This could also explain why we found a correlation between dipping and the hourly urinary sodium excretion ratio only in subjects with cardiovascular risk factors and subjects known to have an older vascular age [45].

In our study, physical activity was not related to the day/night hourly urinary sodium excretion ratio. The absence of a positive correlation does not suggest the need for further similar explorations of the pathophysiological mechanism linking physical activity with blood pressure and sodium metabolism. 

We can therefore affirm that the positive effect of physical activity on blood pressure, especially on the dipping pattern, did not show any link with the pattern of urinary sodium excretion circadian rhythm.

This study has some limitations. Firstly, our data refer to people living in the Italian-speaking part of Switzerland; thus, the results cannot automatically be extrapolated to the general population. Secondly, as this is an observational study, the results cannot be used to define causality. Thirdly, the influence of RSNA was not investigated. Fourth, subjects undergoing diuretic therapy and/or those taking antihypertensive medication were not excluded. Fifth, the level of physical activity was determined by self-report and not measured.

A considerable strength of this study is the rigorous method used to collect urine samples from daytime and nighttime, referring to the individually reported bedtimes and wake-up times. Furthermore, in this investigation, we used a randomly selected representative sample of the population under study, considering all the major elements that could have linked sodium excretion, blood pressure patterns, and physical activity.

## 5. Conclusions

This pilot study shows that while the day/night urinary sodium excretion ratio correlates with the blood pressure dipping pattern, physical activity is unrelated to the circadian rhythm of renal sodium handling. The beneficial effects of physical activity on blood pressure, blood pressure pattern, and cardiovascular health appear thus to be mediated through mechanisms other than the urinary sodium excretion circadian rhythm. These are explorative findings only, but they relativize the need for further investigations on the topic.

## Figures and Tables

**Table 1:** Physical activity level definition.

Sedentary	Little or no physical activity during the day and at work. No heavy physical activity at work.
Partially active	Moderate physical activity. Occasional heavy physical activity at work.
Active	Intense physical activity. Moderate/intense physical activity at work. Almost daily moderate/intense heavy physical activity.

**Table 2:** Patients’ characteristics.

No. of Patients	953
**Demographic characteristics**	
Age, years	49.0 (41–57)
Sex, n (%)	
Female	523 (54.9)
Male	430 (45.1)
**BMI (kg/m^2^)** **Hypertensive drug, n (%)**	24.5 (22.1–27.5) 135 (14.2)
**Blood pressure values**	
Systolic pressure (mmHg)	121 (114–129)
Diastolic pressure (mmHg)	76 (71–83)
Mean BP (mmHg)	91 (85–98)
Blood pressure subgroups, n (%)	
Normotensive, n (%)	746 (78.3)
Mean BP (mmHg)	90 (85–97)
Hypertensive, n (%)	207 (21.7)
Normotensive, n (%)	746 (78.3)
Dipping (%)	
Median (IQR)	8.9 (4.4–12.9)
Dipping ≥ 10%, n (%)	448 (47.0)
Dipping < 10%, n (%)	505 (53.0%)
**Physical activity**	
Walking time day (minutes)	30 (0–600)
Physical activity at work, n (%)	
None	312 (32.7)
Light	364 (38.2)
Moderate	221 (23.2)
Intense	56 (5.9)
Heavy physical activity during the week, n (%)	
None	301 (31.6)
Light	148 (15.5)
Moderate	198 (20.8)
Intense	306 (32.1)
Physical activity level, n (%)	
Sedentary	486 (51.0)
Partially active	210 (22.0)
Active	257 (27.0)
**Sodium metabolism data**	
Hourly ratio of Na excretion (day/night)	1.2 (0.9–1.6)
24 h NaCl intake estimation (g) **	7.9 (5.7–10.4)
Patients with risk factors *, n (%)	550 (57.7)

* Patients were classified at risk when three or more of the following factors were present: smoking, diabetes, previous cardiovascular disease, CKD ≥ 3, LDL ≥ 4.4 mmol/L, hypertension, use of hypolipidemic drugs, use of hypertensive drugs, metabolic syndrome. ** NaCl intake was estimated using the conversion factor: 1 mmol of Na corresponds to 0.0584 g of NaCl.

**Table 3:** Effects of physical activity, NaCl intake, and dipping on circadian rhythm of Na(d)/Na(n) urinary excretion (Total sample).

	Ratio of Na(d)/Na(n)
**Subject Partially active** (baseline: sedentary)	0.01 [−0.11; 0.14]
**Subject Total active** (baseline: sedentary)	0.01 [−0.10; 0.13]
**NaCl intake (g)**	0.01 [−0.01; 0.03]
**Dipping (%)**	0.01 *** [0.01; 0.02]

Statistical significance level: *** *p* < 0.001. Results are reported as β-coefficient and [95%CI]. Sedentary subjects used as baseline.

**Table 4:** Effects of physical activity, NaCl intake, and dipping on circadian rhythm of Na(d)/Na(n) urinary excretion by demographic characteristics.

	(A) Sex		(B) Age Range		
	Female	Male	<40 Years	40–65 Years	>65 Years
**Subject Partially active** (baseline: sedentary)	0.01 [−0.16; 0.18]	0.01 [−0.18; 0.21]	0.02 [−0.29; 0.34]	0.05 [−0.10; 0.20]	0.01 [−0.25; 0.26]
**Subject Total active** (baseline: sedentary)	0.03 [−0.12; 0.19]	−0.01 [−0.20; 0.17]	0.09 [−0.22; 0.42]	0.01 [−0.13; 0.15]	0.20 [−0.04; 0.43]
**NaCl intake (g)**	0.02 [−0.01; 0.04]	0.02 [−0.01; 0.04]	−0.01 [−0.05; 0.02]	0.01 [−0.01; 0.03]	0.06 *** [0.02; 0.09]
**Dipping (%)**	0.01 ** [0.01; 0.02]	0.02 *** [0.01; 0.03]	0.01 [−0.01; 0.03]	0.02 *** [0.01; 0.02]	0.02 ** [0.01; 0.03]

Statistical significance level: *** *p* < 0.001; ** *p* < 0.05. Results are reported as β-coefficient and [95%CI]. Sedentary subjects used as baseline.

**Table 5:** Effects of physical activity, NaCl intake, and dipping on circadian rhythm of Na(d)/Na(n) urinary excretion by clinical characteristics.

	(A) Blood Pressure		(B) Renal Function		(C) Risk Factor *	
	Normal	High	CKD < 3	CKD ≥ 3	No	Yes
**Subject Partially active** (baseline: sedentary)	0.05 [−0.09; 0.20]	−0.14 [−0.43; 0.16]	0.02 [−0.11; 0.15]	−0.06 [−0.88; 0.75]	0.01 [−0.20; 0.22]	0.01 [−0.16; 0.17]
**Subject Total active** (baseline: sedentary)	0.02 [−0.12; 0.15]	0.04 [−0.24; 0.33]	0.02 [−0.11; 0.14]	−0.10 [−0.95; 0.74]	0.08 [−0.09; 0.25]	−0.03 [−0.20; 0.13]
**NaCl intake (g)**	0.01 [−0.01; 0.03]	0.01 [−0.03; 0.04]	0.01 [−0.01; 0.03]	−0.01 [−0.16; 0.13]	0.01 [−0.02; 0.03]	0.02 [−0.01; 0.04]
**Dipping (%)**	0.01 ** [0.01; 0.02]	0.04 *** [0.02; 0.06]	0.01 *** [0.01; 0.02]	0.04 [−0.01; 0.08]	0.01 [−0.01; 0.02]	0.02 *** [0.01; 0.03]

Statistical significance level: *** *p* < 0.001; ** *p* < 0.05. Sedentary subjects used as baseline. * Patients were classified at risk when three or more of the following factors were present: smoking, diabetes, previous cardiovascular disease, CKD ≥ 3, LDL ≥ 4.4 mmol/L, hypertension, use of hypolipidemic drugs, use of hypertensive drugs, metabolic syndrome.

## Data Availability

Study data are obtainable from the authors upon reasonable request.

## References

[1] Lawes C.M., Vander Hoorn S., Rodgers A., International Society of Hypertension (2008). Global burden of blood-pressure-related disease, 2001. Lancet.

[2] Messerli F.H., Williams B., Ritz E. (2007). Essential hypertension. Lancet.

[3] Pickering T.G., Shimbo D., Haas D. (2006). Ambulatory blood-pressure monitoring. N. Engl. J. Med..

[4] Shimbo D., Abdalla M., Falzon L., Townsend R.R., Muntner P. (2015). Role of Ambulatory and Home Blood Pressure Monitoring in Clinical Practice: A Narrative Review. Ann. Intern. Med..

[5] Hansen T.W., Li Y., Boggia J., Thijs L., Richart T., Staessen J.A. (2011). Predictive role of the nighttime blood pressure. Hypertension.

[6] O’Brien E., Sheridan J., O’Malley K. (1988). Dippers and non-dippers. Lancet.

[7] Filippone E.J., Foy A.J., Naccarelli G.V. (2023). Controversies in Hypertension III: Dipping, Nocturnal Hypertension, and the Morning Surge. Am. J. Med..

[8] O’Brien E., Parati G., Stergiou G., Asmar R., Beilin L., Bilo G., Clement D., de la Sierra A., de Leeuw P., Dolan E. (2013). European Society of Hypertension position paper on ambulatory blood pressure monitoring. J. Hypertens..

[9] Verdecchia P., Schillaci G., Guerrieri M., Gatteschi C., Benemio G., Boldrini F., Porcellati C. (1990). Circadian blood pressure changes and left ventricular hypertrophy in essential hypertension. Circulation.

[10] Bianchi S., Bigazzi R., Baldari G., Sgherri G., Campese V.M. (1994). Diurnal variations of blood pressure and microalbuminuria in essential hypertension. Am. J. Hypertens..

[11] Shimada K., Kawamoto A., Matsubayashi K., Ozawa T. (1990). Silent cerebrovascular disease in the elderly. Correlation with ambulatory pressure. Hypertension.

[12] Routledge F., McFetridge-Durdle J. (2007). Nondipping blood pressure patterns among individuals with essential hypertension: A review of the literature. Eur. J. Cardiovasc. Nurs..

[13] Viggiano J., Coutinho D., Clark-Cutaia M.N., Martinez D. (2023). Effects of a high salt diet on blood pressure dipping and the implications on hypertension. Front. Neurosci..

[14] Fukuda M., Goto N., Kimura G. (2006). Hypothesis on renal mechanism of non-dipper pattern of circadian blood pressure rhythm. Med. Hypotheses.

[15] Dong M., McGoldrick M.T., Seid H., Cohen L.P., LaRocca A., Pham P., Thomas S.J., Schwartz J.E., Shimbo D. (2022). The stress, salt excretion, and nighttime blood pressure (SABRE) study: Rationale and study design. Am. Heart J. Plus.

[16] Del Giorno R., Troiani C., Gabutti S., Stefanelli K., Puggelli S., Gabutti L. (2020). Impaired Daytime Urinary Sodium Excretion Impacts Nighttime Blood Pressure and Nocturnal Dipping at Older Ages in the General Population. Nutrients.

[17] Sachdeva A., Weder A.B. (2006). Nocturnal sodium excretion, blood pressure dipping, and sodium sensitivity. Hypertension.

[18] Uzu T., Kimura G., Yamauchi A., Kanasaki M., Isshiki K., Araki S., Sugiomoto T., Nishio Y., Maegawa H., Koya D. (2006). Enhanced sodium sensitivity and disturbed circadian rhythm of blood pressure in essential hypertension. J. Hypertens..

[19] Weinberger M.H. (1996). Salt sensitivity of blood pressure in humans. Hypertension.

[20] DiBona G.F. (2005). Physiology in perspective: The Wisdom of the Body. Neural control of the kidney. Am. J. Physiol. Regul. Integr. Comp. Physiol..

[21] Luft F.C., Weinberger M.H., Fineberg N.S., Miller J.Z., Grim C.E. (1987). Effects of age on renal sodium homeostasis and its relevance to sodium sensitivity. Am. J. Med..

[22] Luft F.C., Fineberg N.S., Miller J.Z., Rankin L.I., Grim C.E., Weinberger M.H. (1980). The effects of age, race and heredity on glomerular filtration rate following volume expansion and contraction in normal man. Am. J. Med. Sci..

[23] Kim Y.G., Moon J.Y., Oh B., Chin H.J., Kim D.K., Park J.H., Shin S.J., Choi B.S., Lim C.S., Lee S.H. (2022). Pressure-Natriuresis Response Is Diminished in Old Age. Front. Cardiovasc. Med..

[24] Salvo F., Lonati C., Berardi M., Errani A.R., Muzzulini C.L., Morganti A. (2017). Nocturnal Blood Pressure Dipping is Abolished in Old-Elderly Hospitalized Patients. High Blood Press. Cardiovasc. Prev..

[25] Bertinieri G., Grassi G., Rossi P., Meloni A., Ciampa M., Annoni G., Vergani C., Mancia G. (2002). 24-hour blood pressure profile in centenarians. J. Hypertens..

[26] Warburton D.E., Nicol C.W., Bredin S.S. (2006). Health benefits of physical activity: The evidence. CMAJ.

[27] WHO (2020). Guidelines on Physical Activity and Sedentary Behaviour.

[28] Cornelissen V.A., Buys R., Smart N.A. (2013). Endurance exercise beneficially affects ambulatory blood pressure: A systematic review and meta-analysis. J. Hypertens..

[29] Sherwood J.J., Inouye C., Webb S.L., Zhou A., Anderson E.A., Spink N.S. (2019). Relationship between physical and cognitive performance in community dwelling, ethnically diverse older adults: A cross-sectional study. PeerJ.

[30] American College of Sports Medicine (2019). Being Active with High Blood Pressure.

[31] Ramirez-Jimenez M., Morales-Palomo F., Moreno-Cabanas A., Alvarez-Jimenez L., Ortega J.F., Mora-Rodriguez R. (2022). Aerobic exercise training improves nocturnal blood pressure dipping in medicated hypertensive individuals. Blood Press. Monit..

[32] Ling C., Diaz K.M., Kretzschmar J., Feairheller D.L., Sturgeon K.M., Perkins A., Veerabhadrappa P., Williamson S.T., Lee H., Grimm H. (2014). Chronic aerobic exercise improves blood pressure dipping status in African American nondippers. Blood Press. Monit..

[33] Mueller P.J. (2007). Exercise training and sympathetic nervous system activity: Evidence for physical activity dependent neural plasticity. Clin. Exp. Pharmacol. Physiol..

[34] Moyers S.A., Hagger M.S. (2023). Physical activity and cortisol regulation: A meta-analysis. Biol. Psychol..

[35] Baffour-Awuah B., Man M., Goessler K.F., Cornelissen V.A., Dieberg G., Smart N.A., Pearson M.J. (2024). Effect of exercise training on the renin-angiotensin-aldosterone system: A meta-analysis. J. Hum. Hypertens..

[36] Dendorfer A., Raasch W., Tempel K., Dominiak P. (1998). Interactions between the renin-angiotensin system (RAS) and the sympathetic system. Basic Res. Cardiol..

[37] Sallsten G., Barregard L. (2021). Variability of Urinary Creatinine in Healthy Individuals. Int. J. Environ. Res. Public Health.

[38] Huang P.L. (2009). A comprehensive definition for metabolic syndrome. Dis. Model. Mech..

[39] Cavero-Redondo I., Martinez-Vizcaino V., Alvarez-Bueno C., Agudo-Conde C., Lugones-Sanchez C., Garcia-Ortiz L. (2019). Metabolic Syndrome Including Glycated Hemoglobin A1c in Adults: Is It Time to Change?. J. Clin. Med..

[40] Harman H. (1976). Modern Factor Analysis.

[41] Rencher C. (2012). Methods of Multivariate Analysis.

[42] Kaiser H.F. (1974). An index of factorial simplicity. Psychometrika.

[43] WHO (2019). Switzerland Physical Activity Factsheet 2019.

[44] Glatz N., Chappuis A., Conen D., Erne P., Pechere-Bertschi A., Guessous I., Forni V., Gabutti L., Muggli F., Gallino A. (2017). Associations of sodium, potassium and protein intake with blood pressure and hypertension in Switzerland. Swiss Med. Wkly..

[45] Zemel M.B., Sowers J.R. (1988). Salt sensitivity and systemic hypertension in the elderly. Am. J. Cardiol..

[46] Jula A. (2024). Sodium—A systematic review for Nordic Nutrition Recommendations 2023. Food Nutr. Res..

